# Development and validation of a population pharmacokinetic model of vancomycin for patients of advanced age

**DOI:** 10.1186/s40780-025-00423-8

**Published:** 2025-03-12

**Authors:** Keisuke Takada, Masaru Samura, Yuki Igarashi, Ayako Suzuki, Tomoyuki Ishigo, Satoshi Fujii, Yuta Ibe, Hiroaki Yoshida, Hiroaki Tanaka, Fumiya Ebihara, Takumi Maruyama, Yukihiro Hamada, Toshiaki Komatsu, Atsushi Tomizawa, Akitoshi Takuma, Hiroaki Chiba, Yusuke Yagi, Yoshifumi Nishi, Yuki Enoki, Kazuaki Taguchi, Koji Tanikawa, Hiroyuki Kunishima, Kazuaki Matsumoto

**Affiliations:** 1 Division of Pharmacodynamics, Keio University Faculty of Pharmacy, 1-5-30 Shibakoen, Minato-Ku, Tokyo, 105-8512 Japan; 2 Department of Pharmacy, Yokohama General Hospital, 2201-5 Kuroganecho, Aoba-Ku, Yokohama City, Kanagawa 225-0025 Japan; 3 Faculty of Pharmaceutical Sciences, Teikyo Heisei University, 4-21-2 Nakano, Nakano-Ku, Tokyo, 164-8530 Japan; 4 Department of Pharmacy, Showa University Fujigaoka Hospital, 1-30 Fujigaoka, Aoba-Ku, Yokohama-Shi, Kanagawa, 227-8501 Japan; 5 Department of Pharmacy, Showa University Hospital, 1-5-8 Hatanodai, Shinagawa-Ku, Tokyo, 142-8666 Japan; 6 Department of Pharmacy, Sapporo Medical University Hospital, 291 Nishi 16-Chome, Minami 1-Jo, Chuo-Ku, Sapporo-Shi, Hokkaido, 060-8543 Japan; 7 Department of Pharmacy, Kyorin University Hospital, 6-20-2 Shinkawa, Mitaka-Shi, Tokyo, 181-8611 Japan; 8 Department of Pharmacy, Tokyo Women’s Medical University Hospital, 8-1 Kawada-Cho, Shinjuku-Ku, Tokyo, 162-8666 Japan; 9 Department of Pharmacy, Kochi Medical School Hospital, 185-1 Okochokohasu, Nankoku-Shi, Kochi, 783-8505 Japan; 10 Department of Pharmacy, Kitasato University Hospital, 1-15-1 Kitasato, Minami-Ku, Sagamihara-Shi, Kanagawa, 252-0375 Japan; 11 Department of Pharmacy, Showa University Northern Yokohama Hospital, 35-1 Chigasaki-Chuo, Tsuzuki-Ku, Yokohama-Shi, Kanagawa, 224-0032 Japan; 12 Department of Hospital Pharmaceutics, School of Pharmacy, Showa University, 1-5-8 Hatanodai, Shinagawa-Ku, Tokyo, 142-8555 Japan; 13 Department of Pharmacy, Tohoku Kosai Hospital, 2-3-11 Kokubuncho, Aoba-Ku, Sendai-Shi, Miyagi, 980-0803 Japan; 14 Center for Pharmacist Education, School of Pharmacy, Nihon University, 7-7-1 Narashinodai, Funabashi-Shi, Chiba, 274-0063 Japan; 15 Department of Infectious Diseases, St. Marianna University School of Medicine Hospital, 2-16-1 Sugao, Miyamae-Ku, Kawasaki City, Kanagawa 216-8511 Japan

**Keywords:** Patients of advanced age, Population pharmacokinetics, Serum albumin, Vancomycin

## Abstract

**Background:**

Population pharmacokinetic (PPK) models of vancomycin (VCM) commonly use creatinine clearance (CLcr) as a covariate for clearance (CL). However, relying on CLcr in patients of advanced age may lead to inaccuracies in estimating VCM clearance. Therefore, this study aimed to develop and validate a new PPK model specifically for patients aged 75 years and older.

**Methods:**

PPK analysis was performed based on the blood concentrations of VCM (*n* = 159 patients). The predictive performance of the developed model was compared with that of previous models using mean absolute error (MAE) and mean squared error (MSE) for another dataset.

**Results:**

The PPK analysis optimized a two-compartment model using CLcr and the Alb levels as covariates at the central compartment of VCM clearance. The final model was as follows: CL (L/h) = 1.96 × (CLcr/3.09) ^0.63^ × (Serum albumin (Alb) /2.3) ^0.22^ × exponential (0.11). Clearance between the central and peripheral compartments (L/h) = 4.86. Central compartment volume of distribution (L) = 31.78. Peripheral compartment volume of distribution (L) = 53.64. The validation study revealed that compared with those of previous models (ranging from 0.67 to 0.79 L/h and from 0.81 to 1.11 (L/h)^2^, respectively), the final model demonstrated the smallest MAE of 0.60 L/h and MSE of 0.65 (L/h)^2^ for patients of advanced age with serum creatinine levels of < 0.6 mg/dL.

**Conclusion:**

The PPK model of VCM for patients of advanced age was optimized by adding the Alb levels and CLcr as covariates for CL. The predictive accuracy of the PPK model for patients with an SCr of < 0.6 mg/dL tended to be higher than those of previous models based just on CLcr. Thus, dosage is suggested to be adjusted based on CLcr and Alb levels for patients with an SCr of < 0.6 mg/dL.

**Supplementary Information:**

The online version contains supplementary material available at 10.1186/s40780-025-00423-8.

## Background

The global population of individuals aged ≥ 65 years is predicted to increase from 9% in 2019 to 16% by 2050, especially the population of individuals aged ≥ 80 years, which is predicted to increase from 143 to 426 million [1]. Patients of advanced age are generally more susceptible to serious infections, including methicillin-resistant *Staphylococcus aureus* (MRSA). Therefore, using the appropriate antimicrobial agent at the correct dosage for these patients is crucial.

Vancomycin (VCM), a glycopeptide antibiotic with activity against gram-positive cocci, has been used for the treatment of methicillin-resistant *Staphylococcus aureus* (MRSA) [2]. The area under the concentration–time curve (AUC) is a predictive parameter that reflects the clinical efficacy and renal toxicity of VCM. VCM achieves clinical efficacy against MRSA at an AUC/minimum inhibitory concentration (MIC) of ≥ 400; however, an AUC of > 600 µg·h/mL has been linked to the risk of renal toxicity [3, 4]. Consequently, AUC-guided dosing has been recommended by the clinical practice guidelines for the therapeutic drug monitoring of VCM [5, 6]. Practical AUC-guided TDM for VCM (PAT) has been used to calculate the AUC in Japan [6]. Several population pharmacokinetic (PPK) models [7, 8] have been developed in Japan; PAT is based on the model proposed by Yasuhara et al. [9].

Approximately 85% of VCM is excreted unchanged through urine by the kidneys; thus, its clearance (CL) is closely related to renal function. Creatinine clearance (CLcr; including serum creatinine [SCr]), which is calculated using the Cockcroft-Gault (CG) equation, is commonly used as a covariate of CL in the VCM model [10]. Age and body weight are also included as covariates in the CG equation [11]. Aging-related renal function decline and physiological changes, such as loss of muscle mass and decrease in serum albumin (Alb) levels, are observed in older adults. This may lead to the over- or underestimation of the CLcr estimated by the CG equation [12–14]. For instance, compared with the actual renal function, the CLcr was underestimated for patients aged ≥ 65 years and those with hypoalbuminemia in the studies conducted by Matsuo et al. and Horio et al. [15, 16]. Yasuhara et al. reported that in previous VCM models, the correlation between measured CLcr and estimated CLcr calculated using the CG equation decreases in patients with SCr < 0.6 mg/dL. Consequently, using CLcr to estimate the CL of VCM may lead to inaccuracies in patients of advanced age [9]. Similar considerations are also necessary for the model proposed by Oda et al., in which CLcr is used as a covariate for CL [8].

Thus, determining the optimal dosage of VCM in patients of advanced age presents several challenges. To address these challenges, we conducted a PPK analysis of VCM in patients aged 75 years and older and developed a new PPK model. We also validated this model and evaluated the optimal dosage regimen using it.

## Methods

A structured three-step-approach, comprising the following steps, was used in this retrospective study:

1. Development of a population pharmacokinetic model for VCM targeting patients of advanced age
2. Validation study of the developed final model for VCM in the target population
3. Optimization of VCM dosing using the final model

### Development of a population pharmacokinetic model for VCM targeting patients of advanced age

The dataset used to develop the PPK model comprised patients who received VCM at the Yokohama General Hospital between August 2016 and September 2024. Patients aged ≥ 75 years and those with a BMI of < 25 kg/m^2^ were included as they constituted the majority population in the Yokohama General Hospital. Patients undergoing dialysis, patients who received VCM for < 3 days and patients with no blood samples with respect to VCM were excluded. Continuous variables are presented as median and range. BMI was calculated using the following equation:

$$\text{weight }(\text{kg})/\text{height}{(\text{m})}^{2}$$

Maintenance dose of VCM was administered based on the condition of the patient at the discretion of the physician. The VCM concentrations were measured using the latex immunoturbidimetric assay. All measurements were performed using DxC 700 AU (Beckman Coulter Inc., CA, USA). Nanopia® TDM Vancomycin (Sekisui Medical Co., Ltd., Tokyo, Japan), the lower detection limit of 2.5 μg/mL, was used as the reagent. For the measurement error of VCM, the coefficient of variation (CV%) in replicate testing for reproducibility was below 15%. The quality control was conducted based on the package insert. The enzymatic method was used to measure the creatinine concentrations.

Phoenix NLME™ (Certara, NJ, USA) was used to perform the PPK analysis. First-order conditional estimation-extended least squares (FOCE-ELS), which is equivalent to the first-order conditional estimation with interaction (FOCE-I) in NONMEM, was used to perform fitting, create plots, and conduct simulations. Phoenix Model with PPK modelling was used to perform the coding. In brief, one- or two-compartment models of the first-order elimination were fitted to the data. Statistical analyses were conducted to evaluate the pharmacokinetic (PK) model. Additive, multiplicative, additive and multiplicative models were evaluated during the examination of the residual variability models (Additional File 1: 1). The shrinkage of the random effect toward the population means was calculated to assess whether the final model was reliable for individual parameters. Subsequently, the covariates were evaluated for central compartment CL and volume of distribution (Vc) as these factors may affect the PK parameters of VCM during the final model estimation. The BMI, Alb levels (modified bromocresol purple method), CLcr, and estimated glomerular filtration rate estimated from creatinine (eGFR) were used to explore the covariates for CL. The CLcr and eGFR were calculated as described in previous studies (Additional File 2: Table 2) [11, 17, 18]. Age, sex, actual body weight, BMI, and Alb levels were used to explore the covariates for Vc. The correlation between covariates was evaluated using the Pearson correlation coefficient and the analysis was conducted using JMP Pro 17.2.0 (JMP Statistical Discovery LLC, USA). Stepwise methods of forward inclusion and backward elimination were used to select the covariates to be included in the model. The objective function value (OFV; -2Log-Likelihood, -2LL) and the Akaike Information Criterion (AIC) were used to evaluate the appropriate compartment model and the final model incorporating the selected covariates for significance. In this study, we defined the accuracy of estimating the CL of VCM in a steady state as clinically significant. When the number of parameters and OFV for CL alone and both Vd and CL were similar, we prioritized incorporating the covariates into CL. The covariates were also evaluated based on the clinical correlations. A decrease of > 3.85 in the OFV was defined as *P* < 0.05 [7]. The omega block variance–covariance matrix was used to evaluate the covariance between the parameters. In addition, a scatterplot matrix and a scatterplot of η were used to evaluate the relevance of the covariates.

The visual predictive check (VPC) method and the bootstrap method (1000 bootstrap runs) were used to verify the goodness of fit of the final VCM model.

### Validation study of the final model for VCM in the target population

The datasets used for validating the developed PPK model comprised patients who had received VCM between September 2020 and December 2023 at eight hospitals encompassing secondary, tertiary, and university hospitals (Sapporo Medical University Hospital, Kyorin University Hospital, Tokyo Women's Medical University Hospital, Showa University Fujigaoka Hospital, Kitasato University Hospital, Tohoku Kosai Hospital, Showa University Northern Yokohama Hospital, and Kochi Medical School Hospital) [19]. Patients aged ≥ 75 years and those with a BMI of < 25 kg/m^2^ were included, with backgrounds similar to those of the PPK analysis group. Those in an intensive care unit or high care unit, and those with an acute renal injury at the time of VCM administration were excluded. The VCM levels were measured using chemiluminescent immunoassay or latex agglutination turbidimetry at three and five facilities, respectively. The lower detection limit, which differed among facilities, ranged from 0.8 to 4 μg/mL. For the measurement error of VCM between facilities, the CV% in replicate testing for reproducibility was approximately 10–15%. For quality control, each facility controlled quality based on the package insert of reagents. For the measurement of Alb, all hospitals adopted the modified bromocresol purple method.

The predictive performance of the final model, compared with that of previous Japanese models, was evaluated using another dataset (Additional File 3: Table 3) [8, 9]. VCM simulation software (PAT), which is commonly used in Japan, was used to define the CL estimated using the Bayesian method from the peak and trough values as a close-to-actual value. This value was compared with the CL estimated from each PPK model [20]. The dose required to achieve an area under the curve in steady state (AUCss) of 500 from each CL was also calculated. The proximity of the maintenance dose calculated using the Bayesian-estimated CL to the actual value was evaluated. The dose was calculated at 250 mg intervals. The mean absolute prediction error (MAE) and mean squared error (MSE) were used to evaluate predictive accuracy (Additional File 4: Table 4). The population was divided into two groups for validation: the first group comprised patients aged ≥ 75 years and the second group comprised patients aged ≥ 75 years with an SCr of < 0.6 mg/dL.

### Optimization of VCM dosing using the final model

The concentration–time profile of VCM in patients was estimated using the Monte Carlo method based on the final PPK model. The AUCss/minimum inhibitory concentration (MIC) ratio was calculated based on the CL, dosage, and MIC in this simulation. The simulations were performed on 15,000 simulated patients for each dosage according to the following procedures. The daily dosages were set at nine doses, namely, 250 mg, 500 mg, 750 mg, 1000 mg, 1250 mg, 1500 mg, 2000 mg, 2500 mg, and 3000 mg. The dosing interval was set as twice a day if the daily dosage was ≥ 1000 mg. The mean and standard deviation of the patients were used to randomly generate covariates for CL in the final PK model with a normal distribution. Furthermore, the CL was calculated using the respective values of the final PK model. The MICs were assigned as follows according to the antimicrobial susceptibility surveillance information provided in the Japanese MRSA guidelines, 2019 edition [21]: 0.25 µg/mL, 0.1%; 0.5 µg/mL, 11.6%; 1.0 µg/mL, 79.3%; and 2.0 µg/mL, 9.0%. The MICs were randomly generated using these ratios.

The probability of target attainment (PTA) was defined as the percentage of patients attaining an AUCss/MIC of ≥ 400 to assess the efficacy of VCM [6]. This criterion was used to calculate the PTA for each CL and dosage. All analyses were conducted using Microsoft 365 Excel (Microsoft, Redmond, WA, USA).

We defined a PTA of at least 85% with an AUCss/MIC of ≥ 400 as the optimal dosage to prevent a high incidence of AKI. PTA was calculated using Monte Carlo simulation based on the final model, which incorporated covariates. The probability of achieving an AUCss of > 600 µg·h/mL was also calculated to assess the safety of VCM. Subsequently, the probability of achieving an AUCss of > 600 µg·h/mL was classified as mild risk (< 10%), moderate risk (10–25%), and severe risk (> 25%) according to the definition of risk classification of acute kidney injury of VCM [22].

## Results

### Patient characteristics

Table 1 presents the characteristics of the patients in the modeling (*n* = 159) and validation (*n* = 133) datasets. The median age in the modeling and validation datasets was 84 (75–99) years and 81 (75–101) years, respectively. Individuals aged ≥ 85 years accounted for 49.7% and 35.4% of the patients in the modeling and validation datasets, respectively. The median BMI in the modeling and validation datasets was 18.6 (11.0–24.8) kg/m^2^ and 20.0 (11.9–24.9) kg/m^2^, respectively. Individuals with a BMI of < 18.5 kg/m^2^ accounted for 48.4% and 28.6% of the patients in the modeling and validation datasets, respectively. The median Alb level in the modeling and validation datasets was 2.3 (1.2–4.2) g/dL and 2.5 (1.2–4.4) g/dL, respectively. Individuals with a median Alb level of < 3.0 g/dL accounted for 90.6% and 78.2% of the patients in the modeling and validation datasets, respectively. The median CLcr in the modeling and validation datasets was 3.06 (0.58–7.26) L/h [51.0 (9.7–121.0) mL/min] and 3.05 (0.46–6.64) L/h [50.9 (7.7–110.6) mL/min], respectively. The daily maintenance dose of VCM in the modeling and validation datasets was commonly 1000–2000 mg (67.9–73.7%).

**Table 1:** Patient characteristics

Characteristics	Modeling dataset (*n* = 159) Number (%) or median (min–max)	Validation dataset (*n* = 133) Number (%) or median (min–max)
Age (years)	84 (75–99)	81 (75–101)
75–84	80 (50.3)	86 (64.7)
85–94	73 (45.9)	46 (34.6)
≥ 95	6 (3.8)	1 (0.8)
Sex (male/female)	92 (57.9)/67 (42.1)	69 (51.9)/64 (48.1)
Height (cm)	158 (135–180)	157 (137–182)
Body weight (kg)	47 (26–70)	49 (29–70)
Body Mass Index (kg/m^2^)	18.6 (11.0–24.8)	20.0 (11.9–24.9)
< 18.5	77 (48.4)	38 (28.6)
Serum creatinine (mg/dL)	0.64 (0.22–3.00)	0.69 (0.24–5.68)
< 0.60	67 (42.1)	40 (30.1)
Estimated creatinine clearance (mL/min)	51.5 (9.7–121.2)	50.9 (7.7–110.6)
≤ 50.0	75 (47.2)	63 (47.4)
The glomerular filtration rate estimated from creatinine (mL/min)	65.5 (12.4–159.1)	68.4 (7.5–195.4)
≤ 50.0	45 (28.3)	25 (18.8)
Serum albumin (g/dL)	2.3 (1.2–4.2)	2.5 (1.2–4.4)
< 2.0	31 (19.5)	19 (14.3)
2.0–2.9	113 (71.1)	85 (63.9)
3.0–3.5	9 (5.7)	19 (14.3)
≥ 3.6	6 (3.8)	10 (7.5)
Daily maintenance dose of Vancomycin (mg/day)	1500 (250–3000)	1500 (300–3000)
< 1000	44 (27.7)	31 (23.3)
1000–2000	108 (67.9)	98 (73.7)
> 2000	7 (4.4)	4 (3.0)

*BMI* Body Mass Index, *CLcr* estimated creatinine clearance, *eGFR* the glomerular filtration rate estimated from creatinine, *VCM* Vancomycin

### Development of a population pharmacokinetic model for VCM targeting older patients

**Fig. 1:**
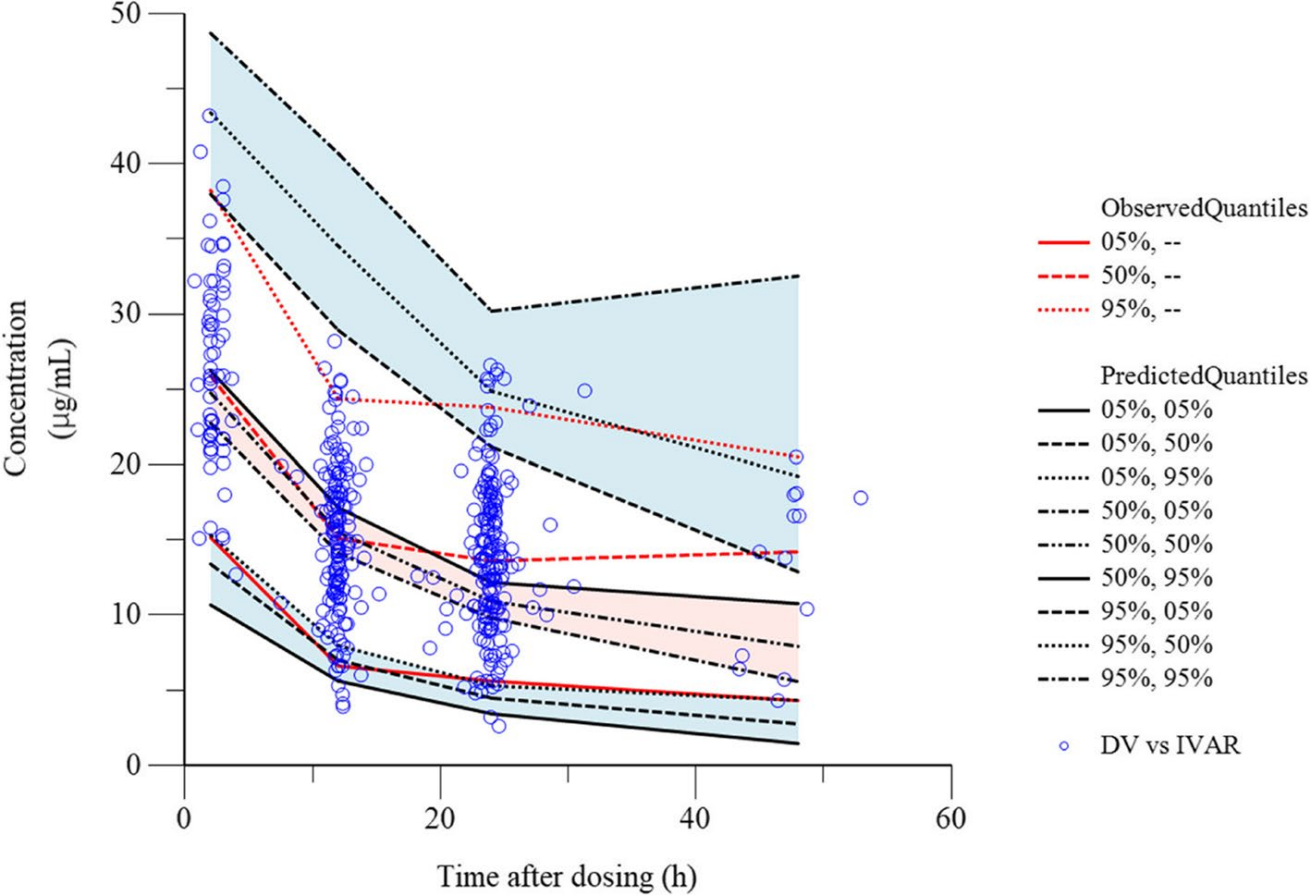
Visual predictive check plot of vancomycin. Visual predictive check plot of the vancomycin concentration versus time for the covariate model. Concentration vs. Time after dosing. Observed vs. individual predicted concentrations

The PPK analysis included 417 samples (peak concentration: 65 samples; trough concentration: 352 samples) obtained from 159 patients who satisfied the inclusion criteria. No samples with concentrations below the limit of quantitation were included in the analysis. Tables 2 and 3 present the results of the exploration of the covariates using the stepwise method and the results of the final and bootstrap method models, respectively. A two-compartment model was optimal for VCM. Thus, the PPK model was optimized by adding the Alb levels and CLcr as covariates for CL. The Pearson correlation coefficient in Alb and CLcr was -0.12 (95% confidence interval: -0.27–0.03, *p* = 0.12). Additional File 5: Fig. 1 presents the diagnostic plots for VCM. Notably, the individual predicted concentrations exhibited good correlation with the observed concentrations in the final model (Additional File 5: Fig. 1). The typical values, standard errors, coefficients of variation, and 95% confidence intervals for the final model of VCM were generally similar to those for the bootstrap method (Table 3). Furthermore, the observed and predicted concentrations for VPC were close to the fifth, 50th, and 95th percentiles (Fig. 1). Conditionally weighted residual plots were generally plotted on a scale of -3 to + 3 and distributed evenly on both sides of the x-axis (Additional File 5: Fig. 1). Based on these results, the final model of VCM was optimized as follows: CL (L/h) = 1.96 × (CLcr/3.09) ^0.63^ × (Alb/2.3) ^0.22^ × exp.(0.11). Clearance between the central and peripheral compartments (Q) (L/h) = 3.24. Vc (L) = 31.78. The peripheral compartment volume of distribution (Vp) (L) = 53.64. The median and range of CL for the PPK parameters in individual patients was 1.96 L/h (1.84–2.08). Moreover, Vc was a fixed value, with a mean value and 95% confidence interval of 31.78 L (24.19–39.38) (Table 3).

**Table 2:** Stepwise search for covariates in the final model of vancomycin concentrations

Model	Description	−2LL	Δ -2LL	AIC	n-parameter
1	Base model	2431.60	–	2445.60	7
2	Central clearance on estimated glomerular filtration rate unadjusted for body surface area	2355.51	76.09^a^	2371.51	8
3	Central clearance on estimated creatinine clearance	2331.23	100.37^a^	2347.23	8
4	Central clearance on estimated creatinine clearance and Serum albumin level	2326.11	5.11^b^	2344.11	9

*-2LL* -2 log likelihood, *AIC* Akaike information criterion, eGFR estimated glomerular filtration rate unadjusted for body surface area, CLcr estimated creatinine clearance

^a^Compared with model 1

^b^Compared with model 3

**Table 3:** Final population pharmacokinetic parameter estimates of vancomycin and bootstrap validation

Parameter	Final model				Bootstrap method (*n* = 1000)			
	Estimate	SE	CV (%)	95% CI	Estimate	SE	CV (%)	95% CI
Confidence interval (L/h) = θ_1_ × (CLcr/3.09) ^θ2^ × (Alb/2.3) ^θ3^ × exp. (ηCL)								
typical value of apparent clearance (L/h)	1.96	0.06	3.11	1.84 – 2.08	1.94	0.10	4.92	1.73 – 2.11
exponent for CLcr as covariate	0.63	0.06	9.72	0.51 – 0.76	0.64	0.07	10.47	0.50 – 0.77
exponent for Alb as a covariate for CL	0.22	0.09	41.75	0.03 – 0.40	0.21	0.11	52.68	0.01 – 0.43
intercompartmental clearance of vancomycin (L/h) = θ_4_								
typical value of apparent intercompartmental clearance (L/h)	4.86	0.91	18.63	3.08 – 6.64	4.57	1.49	32.70	1.00 – 7.21
volume of distribution of vancomycin in the central compartment (L) = θ_5_								
typical value of apparent volume of distribution (L)	31.78	3.86	12.16	24.19 – 39.38	32.23	6.06	18.80	20.88 – 46.86
volume of distribution of vancomycin in the peripheral compartment (L) = θ_6_								
typical value of apparent volume of distribution in the peripheral compartment (L)	53.64	4.22	7.86	45.36 – 61.93	55.26	7.60	13.75	45.20 – 73.38

*SE* Standard error, *CV* Coefficient of variation, *CI* Confidence interval, θ, population mean, θ_1_, typical value of apparent clearance, θ_2_, exponent for CLcr as covariate, θ_3_, exponent for Alb as a covariate for CL; θ_4_, typical value of apparent intercompartmental clearance, θ_5_, typical value of apparent volume of distribution, θ_6_, typical value of apparent volume of distribution in the peripheral compartment, η, random variable, which is normally distributed with mean 0 and variance ω^2^, ηCL = 0.11, CL, clearance of vancomycin, Q, intercompartmental clearance of vancomycin, Vc, volume of distribution of vancomycin in the central compartment, Vp, volume of distribution of vancomycin in the peripheral compartment

### Validation study of the developed final model for VCM

**Fig. 2:**
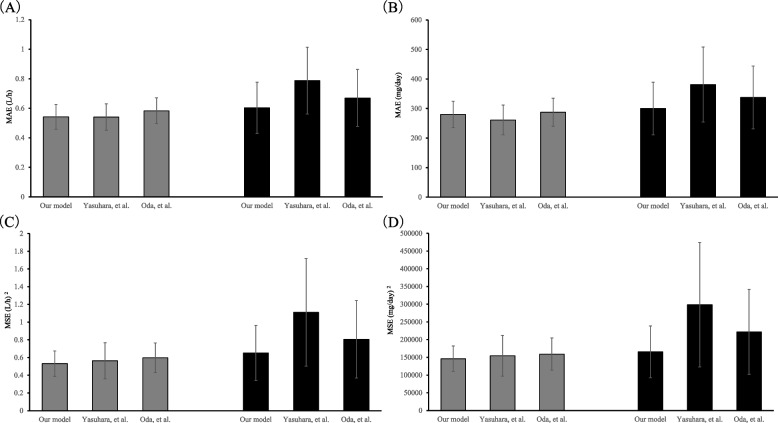
Validation of the final model. **A** and **B**. The height of the bars indicates the mean absolute prediction error (MAE) of CL and the dosage at 250 mg intervals achieving an area under the concentration–time curve at a steady state of 500, respectively. **C** and **D**. The height of the bars indicates the mean squared error (MSE) of CL and the dosage at 250 mg intervals achieving an area under the concentration–time curve at a steady state of 500, respectively. The grey and black bars represent patients aged ≥ 75 years and patients aged ≥ 75 years with an SCr of < 0.6 mg/dL, respectively. The error bars represent their corresponding 95% confidence intervals

The MAE/MSE of CL and dosage achieving AUCss of 500 µg·h/mL in the validation datasets are plotted in Fig. 2 as representative mean [95% confidence interval (CI)] values. The MAE and MSE of CL for patients aged ≥ 75 years in the developed model were 0.54 L/h [95% CI 0.46–0.63 L/h] and 0.53 (L/h)^2^ [95% CI 0.39–0.68 (L/h)^2^], respectively. The MAE and MSE of the dosage achieving an AUCss of 500 µg·h/mL were 280.08 mg/day (95% CI 235.28–324.87 mg/day) and 146,146.62 (mg/day)^2^ [95% CI 110,399.18–181,894.05 (mg/day)^2^], respectively. Comparison with previous models revealed similarity. The MAE and MSE of CL for patients aged ≥ 75 years with SCr < 0.6 mg/dL in the developed model were 0.60 L/h (95% CI 0.43–0.77 L/h) and 0.65 (L/h)^2^ [95% CI 0.34–0.96 (L/h)^2^], respectively. The MAE and MSE of the dosage achieving an AUCss of 500 µg·h/mL were 300.00 mg/day (95% CI 210.93–389.07 mg/day) and 165,625.00 (mg/day)^2^ [95% CI 92,560.27–238,689.73 (mg/day)^2^], respectively. Among the developed and previous models, the lowest MAE and MSE were exhibited by the model proposed herein.

### Optimization of VCM dosing using the final model

The CL determined based on the final model using CLcr and Alb levels as covariates and the range of values for the patients in this study generally ranged from 0.5 L/h to 3.75 L/h (Additional File 6: Table 5). The daily dosage of VCM required to achieve a PTA of ≥ 85% ranged from 250 to 2000 mg (Additional File 7: Table 6). Some minimum dosages for each CL that achieved a PTA of ≥ 85% had a probability of AUCss > 600 µg·h/mL of 25% or higher. (Additional File 8: Table 7). The optimal daily dosage to achieve a PTA of ≥ 85% based on an AUCss/MIC of ≥ 400 for Alb levels of 1.5 g/dL and 2.0–3.5 g/dL was 500–1500 mg/day and 750–1500 mg/day, respectively, when the CLcr was set as 20–85 mL/min considering the quartile range of this study patients. The optimal daily dosage of VCM for Alb levels of 1.5 g/dL and 2.0–3.5 g/dL was 750 mg and 1000 mg, respectively, when a CLcr of 40 mL/min was set as the relatively common renal function (Table 4). The minimum VCM daily dosage to achieve an AUCss/MIC of ≥ 400 at ≥ 85% for CL of 1.25–2.25 L/h and CL 2.5–3.25 L/h (for MIC 0.5 µg/mL) was 500 mg and 750 mg, respectively. Furthermore, the dosages for CL of 1.25–1.75 L/h, CL 2.0–2.25 L/h, 2.5 L/h, and CL 2.75–3.25 L/h (for MIC 1.0 µg/mL) were 750 mg, 1000 mg, 1250 mg, and 1500 mg, respectively (Additional File 9: Fig. 2). The constructed nomogram revealed that an AUCss of > 600 µg·h/mL was observed in 41.5% of some CLcr and Alb levels (Additional File 10: Table 8) (Table 4).

**Table 4:** Optimal dose of vancomycin based on the creatinine clearance and serum Albumin levels

serum albumin (g/dL)	Daily maintenance dose (mg/day; if ≥ 1000 mg is divided into two doses)													
	Creatinine clearance (L/h) [Creatinine clearance (mL/min)]													
	1.2 [20]	1.5 [25]	1.8 [30]	2.1 [35]	2.4 [40]	2.7 [45]	3.0 [50]	3.3 [55]	3.6 [60]	3.9 [65]	4.2 [70]	4.5 [75]	4.8 [80]	5.1 [85]
1.5	500	750	750	750	750	1000	1000	1000	1000	1250	1250	1250	1500	1500
2.0	750	750	750	750	1000	1000	1000	1000	1250	1250	1500	1500	1500	1500
2.5	750	750	750	750	1000	1000	1000	1250	1250	1500	1500	1500	1500	1500
3.0	750	750	750	1000	1000	1000	1250	1250	1500	1500	1500	1500	1500	1500
3.5	750	750	750	1000	1000	1000	1250	1500	1500	1500	1500	1500	1500	1500

We estimated the optimal maintenance dose of vancomycin that achieved a probability of target attainment ≥ 85% at steady state to achieve the target AUCss/MIC ≥ 400

*Alb* serum albumin, *VCM* Vancomycin, *CLcr* Creatinine clearance, *AUCss* Area under the concentration–time curve of vancomycin from 0 to 24 h at steady state, *MIC* Minimum inhibitory concentration

## Discussion

The present study revealed that the prediction accuracy of VCM CL in PPK analysis for patients of advanced age is higher when CLcr and Alb levels are used as covariates. Furthermore, the prediction accuracy was exceptionally high among patients with an SCr of < 0.6 mg/dL.

The median CL was 1.96 L/h and 2.45–4.73 L/h in the present study and previous PPK analyses involving Japanese patients, respectively [9, 23, 24]. In the modeling dataset, individuals aged ≥ 85 years accounted for 49.7%, individuals with a CLcr of < 3.00 L/h (50 mL/min) accounted for 47.2%, individuals with a BMI of < 18.5 kg/m^2^ accounted for 48.4%, and individuals with Alb levels of < 3.0 g/dL accounted for 90.6%. In contrast, individuals with a mean age of 44.5–78.3 years, mean CLcr of 3.38–4.81 L/h (56.3–80.2 mL/min), mean BMI of 23.4 kg/m^2^, mean Alb levels of 2.9 g/dL were included in previous PPK analyses [9, 23, 24]. Matsuo et al. reported that the CLcr decreases with aging [15]. Chang et al. reported that compared with that observed in individuals with a BMI of ≥ 18.5 kg/m^2^, the eGFR was significantly lower in individuals with a BMI of < 18.5 kg/m^2^ [25]. Furthermore, a significant positive correlation was observed between the Alb levels and eGFR in individuals with stage 2–5 chronic kidney disease [26]. Thus, compared with previous studies, the present study included more patients with impaired renal function and those with lower CLcr.

The covariate CL of VCM only incorporated the CLcr estimated using the CG equation in previous studies [9, 23, 24]. However, the present study revealed that the model with the Alb levels added to the CLcr estimated using the CG equation was optimal. A limitation of the estimating equation for renal function using SCr and age is that the renal function is significantly underestimated in patients aged ≥ 65 years, those with a BMI of < 25 kg/m^2^, and those with Alb levels of < 4.0 g/dL [15, 16, 27]. Several patients with these characteristics were included in the present study. Notably, incorporating only CLcr as a covariate in the CL of VCM may decrease accuracy. However, Matsuo et al. reported that the addition of the Alb levels to the estimating equation for renal function improved the accuracy of estimating the GFR of the Japanese patients included in their study, among whom 54% had stage ≥ 3 chronic kidney disease and mean Alb levels of 3.8 g/dL [17]. Levey et al. conducted a study involving patients with chronic kidney disease with a mean GFR of 2.39 L/h/1.73 m^2^ (39.8 mL/min/1.73 m^2^) and mean Alb levels of 4.0 g/dL and reported that the accuracy of estimating GFR was improved when SCr and the Alb levels were included in the estimation of renal function [28]. The mean age of the patient populations in these two studies ranged from 50.6 to 51.4 years [17, 28]. Further evaluation is needed to determine their applicability in estimating renal function and VCM clearance for advanced-age patients. Baumgartner, R. N. et al. investigated the relationship between Alb and muscle mass in a study of 275 individuals aged 60–95 years [29]. In the study, Alb was measured and muscle mass was assessed using dual-energy X-ray absorptiometry. Their analysis revealed a positive correlation between Alb and muscle mass. Ohtani et al. also examined the factors that affect the measured CLcr in bedridden patients of advanced age and showed that Alb was a significant factor, along with the estimated CLcr, body fat mass (BFM), and triceps skinfold thickness (TSF) [30]. However, collecting BFM and TSF was impossible in the present retrospective study. As a result, we reasoned that including Alb as a covariate for CL in the PPK model of VCM in advanced-age patients with decreasing muscle mass would be clinically significant. When the CLcr was set as 5.1 L/h (85.0 mL/min), the CL of VCM determined using the model equation developed in the present study for Alb levels of 1.5 g/dL and 3.5 g/dL was 2.73 L/h (45.5 mL/min) and 3.29 L/h (54.8 mL/min), respectively. Thus, a 2.0 g/dL decrease in the Alb levels would result in a difference of 0.56 L/h (9.3 mL/min) for CL of VCM even if CLcr was maintained. Thus, compared with CLcr alone, incorporating CLcr and the Alb levels as covariates may have accurately corrected CL in the modeling dataset population when the Alb levels decreased.

The lower MAE and MSE achieved by the final model, particularly for CL of VCM and dosage achieving an AUCss of 500 µg·h/mL in patients with an SCr of < 0.6 mg/dL, indicate improved accuracy. Discrepancies between the estimated and actual CLcr was observed in patients with an SCr of < 0.6 mg/dL in the study conducted by Yasuhara et al., leading to prediction errors during the estimation of CL of VCM based solely on CLcr [9]. The final model achieved greater precision in estimating CL of VCM in this population following the incorporation of the Alb levels, thereby reducing the likelihood of prediction errors. This suggests that the developed model in this study applies to patients of advanced age, particularly for patients aged ≥ 75 years with SCr < 0.6 mg/dL.

The nomogram revealed that the daily dosage of 750 mg achieved ≥ 85% PTA resulting in an AUCss/MIC of ≥ 400 for a CLcr of 2.4 L/h (40.0 mL/min) and Alb levels of 1.5 g/dL. This dosage is lower than that recommended by the guideline (1000 mg) [6]. The recommended dosage in the present study was all PTA < 85% for VCM MIC = 2 μg/mL. The meta-analysis by Samura et al. also evaluated the efficacy of VCM and daptomycin (DAP) in patients with MRSA bacteremia with VCM > 1 μg/mL [31]. DAP was significantly more effective. We recommend using other drugs for MRSA infections with a VCM MIC of 2 μg/mL based on these findings. This may be attributed to the decrease in the CL of VCM owing to the Alb levels correcting the CL in the final model. In addition, an AUCss of > 600 µg·h/mL was calculated > 25% for some CLcr and Alb. Therefore, early TDM and reassessment, as well as a one-step dosage reduction for patients at risk of kidney injury, should be considered for patients in whom an AUCss of > 600 µg·h/mL is achieved at ≥ 25%.

There are certain limitations to this study. First, the CLcr of the patients included in the present study ranged from 0.58 L/h (9.7 mL/min) to 7.27 L/h (121.2 mL/min). Furthermore, only a few patients were close the upper and lower limits. Second, the proposed model was validated using 40 cases with an SCr of < 0.6 mg/dL in the validation dataset, although the number of cases was limited. Finally, we defined a PTA of ≥ 85% as the optimal dosage to prevent a high incidence of AKI. Therefore, it is necessary to consider early TDM or using a one-step higher dosage in patients with severe infections.

## Conclusions

The PPK model of VCM for patients of advanced age was optimized by adding the Alb levels and CLcr as covariates for CL. Notably, the predictive accuracy of the proposed PPK model for patients with a SCr of < 0.6 mg/dL tended to be higher than those of previous models that relied on CLcr alone. These findings suggest that the dosage for patients with a SCr of < 0.6 mg/dL should be adjusted based on the CLcr and Alb levels.

## Supplementary Information

Additional file 1.

Additional file 2.

Additional file 3.

Additional file 4.

Additional file 5.

Additional file 6.

Additional file 7.

Additional file 8.

Additional file 9.

Additional file 10.

## Data Availability

No datasets were generated or analysed during the current study.

## Abbreviations

VCM: Vancomycin
MRSA: Methicillin-resistant Staphylococcus aureus
AUC: Area under the concentration–time curve
MIC: Minimum inhibitory concentration
PAT: Practical AUC-guided TDM for VCM
PPK: Population pharmacokinetic
CL: Clearance
CLcr: Creatinine clearance
SCr: Serum creatinine level
CysC: Cystatin C
CG: Cockcroft–Gault
Alb: Serum albumin level
BMI: Body mass index
eGFR: Estimated glomerular filtration rate estimated from creatinine
Vc: Central compartment volume of distribution
OFV: Objective function value
LL: Log-Likelihood
AIC: Akaike Information Criterion
VPC: Visual predictive check
AUCss: Area under the concentration–time curve at steady state
MAE: Mean absolute prediction error
MSE: Mean standard error
PTA: Probability of target attainment
Q: Clearance between the central and peripheral compartments
Vp: Peripheral compartment volume of distribution
CI: Confidence interval
DAP: Daptomycin under

## References

[1] United Nations Department of Global Communications. PRESS RELEASE. Growing at a slower pace, world population is expected to reach 9.7 billion in 2050 and could peak at nearly 11 billion around 2100: UN Report https://population.un.org/wpp/Publications/Files/WPP2019_PressRelease_EN.pdf. Accessed 11 Oct 2023.

[2] Sorrell TC, Packham DR, Shanker S, Foldes M, Munro R. Vancomycin therapy for methicillin-resistant staphylococcus aureus. Ann Intern Med. 1982;97:344–50. 10.7326/0003-4819-97-3-344. 7114631 10.7326/0003-4819-97-3-344

[3] Moise-Broder PA, Forrest A, Birmingham MC, Schentag JJ. Pharmacodynamics of vancomycin and other antimicrobials in patients with Staphylococcus aureus lower respiratory tract infections. Clin Pharmacokinet. 2004;43:925–42. 10.2165/00003088-200443130-00005. 15509186 10.2165/00003088-200443130-00005

[4] Tsutsuura M, Moriyama H, Kojima N, Mizukami Y, Tashiro S, Osa S, et al. The monitoring of vancomycin: a systematic review and meta-analyses of area under the concentration-time curve-guided dosing and trough-guided dosing. BMC Infect Dis. 2021;21:153. 10.1186/s12879-021-05858-6. 33549035 10.1186/s12879-021-05858-6 PMC7866743

[5] Rybak MJ, Le J, Lodise TP, Levine DP, Bradley JS, Liu C, et al. Therapeutic monitoring of vancomycin for serious methicillin-resistant Staphylococcus aureus infections: a revised consensus guideline and review by the American Society of Health-System Pharmacists, the Infectious Diseases Society of America, the Pediatric Infectious Diseases Society, and the Society of Infectious Diseases Pharmacists. Am J Health Syst Pharm. 2020;77:835–64. 10.1093/ajhp/zxaa036. 32191793 10.1093/ajhp/zxaa036

[6] Matsumoto K, Oda K, Shoji K, Hanai Y, Takahashi Y, Fujii S, et al. Clinical practice guidelines for therapeutic drug monitoring of vancomycin in the framework of model-informed precision dosing: A consensus review by the Japanese Society of Chemotherapy and the Japanese Society of Therapeutic Drug Monitoring. Pharmaceutics. 2022;14. 10.3390/pharmaceutics14030489. 10.3390/pharmaceutics14030489 PMC8955715 35335866

[7] Yamamoto M, Kuzuya T, Baba H, Yamada K, Nabeshima T. Population pharmacokinetic analysis of vancomycin in patients with gram-positive infections and the influence of infectious disease type. J Clin Pharm Ther. 2009;34:473–83. 10.1111/j.1365-2710.2008.01016.x. 19583681 10.1111/j.1365-2710.2008.01016.x

[8] Oda K, Matsumoto K, Shoji K, Shigemi A, Kawamura H, Takahashi Y, et al. Validation and development of population pharmacokinetic model of vancomycin using a real-world database from a nationwide free web application. J Infect Chemother. 2024;30:1244–51. 10.1016/j.jiac.2024.05.014. 38825002 10.1016/j.jiac.2024.05.014

[9] Yasuhara M, Iga T, Zenda H, Okumura K, Oguma T, Yano Y, et al. Population pharmacokinetics of vancomycin in Japanese adult patients. Ther Drug Monit. 1998;20:139–48. 10.1097/00007691-199804000-00003. 9558127 10.1097/00007691-199804000-00003

[10] Marsot A, Boulamery A, Bruguerolle B, Simon N. Vancomycin: a review of population pharmacokinetic analyses. Clin Pharmacokinet. 2012;51:1–13. 10.2165/11596390-000000000-00000. 22149255 10.2165/11596390-000000000-00000

[11] Cockcroft DW, Gault MH. Prediction of creatinine clearance from serum creatinine. Nephron. 1976;16:31–41. 10.1159/000180580. 1244564 10.1159/000180580

[12] Huang S-M. Atkinson’s principles of clinical pharmacology. 4th ed. London, England: Academic Press; 2022.

[13] Janssen I, Heymsfield SB, Wang ZM, Ross R. Skeletal muscle mass and distribution in 468 men and women aged 18–88 yr. J Appl Physiol. 2000;89:81–8. 10.1152/jappl.2000.89.1.81. 10904038 10.1152/jappl.2000.89.1.81

[14] Gom I, Fukushima H, Shiraki M, Miwa Y, Ando T, Takai K, et al. Relationship between serum albumin level and aging in community-dwelling self-supported elderly population. J Nutr Sci Vitaminol (Tokyo). 2007;53:37–42. 10.3177/jnsv.53.37. 17484377 10.3177/jnsv.53.37

[15] Matsuo M, Yamagishi F. Age-dependent error in creatinine clearance estimated by Cockcroft-Gault equation for the elderly patients in a Japanese hospital: a cross-sectional study. J Anesth. 2019;33:155–8. 10.1007/s00540-018-2596-3. 30603825 10.1007/s00540-018-2596-3

[16] Horio M, Imai E, Yasuda Y, Watanabe T, Matsuo S. Lower serum albumin level is associated with higher fractional excretion of creatinine. Clin Exp Nephrol. 2014;18:469–74. 10.1007/s10157-013-0841-5. 23877710 10.1007/s10157-013-0841-5

[17] Matsuo S, Imai E, Horio M, Yasuda Y, Tomita K, Nitta K, et al. Revised equations for estimated GFR from serum creatinine in Japan. Am J Kidney Dis. 2009;53:982–92. 10.1053/j.ajkd.2008.12.034. 19339088 10.1053/j.ajkd.2008.12.034

[18] Du Bois D, Du Bois EF. A formula to estimate the approximate surface area if height and weight be known. 1916. Nutrition. 1989;5:303–11 discussion 12-3. 2520314

[19] Suzuki A, Samura M, Ishigo T, Fujii S, Ibe Y, Yoshida H, et al. Identification of patients who require two-point blood sampling for the peak and trough values rather than one-point blood sampling for the trough value for the evaluation of AUC of vancomycin using Bayesian estimation. Pharm Res. 2024;41:2161–71. 10.1007/s11095-024-03781-4. 39433691 10.1007/s11095-024-03781-4

[20] Oda K, Hashiguchi Y, Kimura T, Tsuji Y, Shoji K, Takahashi Y, et al. Performance of area under the concentration-time curve estimations of vancomycin with limited sampling by a newly developed web application. Pharm Res. 2021;38:637–46. 10.1007/s11095-021-03030-y. 33782837 10.1007/s11095-021-03030-y

[21] Committee of practical guidelines for the treatment of MRSA infections. Practical guidelines for the management and treatment of infections caused by MRSA, 2019 Edition. Japanese society of chemotherapy (Tokyo, Japan) / The Japanese association for infectious diseases (Tokyo, Japan) 2019. http://www.chemotherapy.or.jp/guideline/guideline_mrsa_2019.pdf. Accessed 18 Jul 2023. Japanese.

[22] Miyai T, Imai S, Kashiwagi H, Sato Y, Kadomura S, Yoshida K, et al. A risk prediction flowchart of vancomycin-induced acute kidney injury to use when starting vancomycin administration: a multicenter retrospective study. Antibiotics (Basel). 2020;9. 10.3390/antibiotics9120920. 10.3390/antibiotics9120920 PMC7766575 33352848

[23] Ducharme MP, Slaughter RL, Edwards DJ. Vancomycin pharmacokinetics in a patient population: effect of age, gender, and body weight. Ther Drug Monit. 1994;16:513–8. 10.1097/00007691-199410000-00013. 7846752 10.1097/00007691-199410000-00013

[24] Zhou Y, Gao F, Chen C, Ma L, Yang T, Liu X, et al. Development of a population pharmacokinetic model of vancomycin and its application in Chinese geriatric patients with pulmonary infections. Eur J Drug Metab Pharmacokinet. 2019;44:361–70. 10.1007/s13318-018-0534-2. 30506225 10.1007/s13318-018-0534-2 PMC6520475

[25] Chang TJ, Zheng CM, Wu MY, Chen TT, Wu YC, Wu YL, et al. Author correction: relationship between body mass index and renal function deterioration among the Taiwanese chronic kidney disease population. Sci Rep. 2020;10:2822. 10.1038/s41598-020-59783-w. 32054951 10.1038/s41598-020-59783-w PMC7018810

[26] Cheng T, Wang X, Han Y, Hao J, Hu H, Hao L. The level of serum albumin is associated with renal prognosis and renal function decline in patients with chronic kidney disease. BMC Nephrol. 2023;24:57. 10.1186/s12882-023-03110-8. 36922779 10.1186/s12882-023-03110-8 PMC10018824

[27] Verhave JC, Fesler P, Ribstein J, du Cailar G, Mimran A. Estimation of renal function in subjects with normal serum creatinine levels: influence of age and body mass index. Am J Kidney Dis. 2005;46:233–41. 10.1053/j.ajkd.2005.05.011. 16112041 10.1053/j.ajkd.2005.05.011

[28] Levey AS, Bosch JP, Lewis JB, Greene T, Rogers N, Roth D. A more accurate method to estimate glomerular filtration rate from serum creatinine: a new prediction equation. Modification of Diet in Renal Disease Study Group. Ann Intern Med. 1999;130:461–70. 10.7326/0003-4819-130-6-199903160-00002. 10075613 10.7326/0003-4819-130-6-199903160-00002

[29] Baumgartner RN, Koehler KM, Romero L, Garry PJ. Serum albumin is associated with skeletal muscle in elderly men and women. Am J Clin Nutr. 1996;64:552–8. 10.1093/ajcn/64.4.552. 8839499 10.1093/ajcn/64.4.552

[30] Otani T, Kase Y, Kunitomo K, Shimooka K, Naoe M, Yamamoto H, Kawazoe K, Sato Y, Yamauchi A. Novel formula using triceps skinfold thickness to revise the Cockcroft-Gault equation for estimating renal function in Japanese bedridden elderly patients. J Med Invest. 2018;65:195–202. 10.2152/jmi.65.195. 30282860 10.2152/jmi.65.195

[31] Samura M, Kitahiro Y, Tashiro S, Moriyama H, Hamamura Y, Takahata I, Kawabe R, Enoki Y, Taguchi K, Takesue Y, Matsumoto K. Efficacy and safety of daptomycin versus vancomycin for bacteremia caused by methicillin-resistant staphylococcus aureus with vancomycin minimum inhibitory concentration > 1 µg/mL: a systematic review and meta-analysis. Pharmaceutics. 2022;27(14):714. 10.3390/pharmaceutics14040714. 10.3390/pharmaceutics14040714 PMC9032134 35456548

