# Supplementary material for: Development and validation of a population pharmacokinetic model of vancomycin for patients of advanced age
Source: J Pharm Health Care Sci. 2025 Mar 12;11:18. doi: 10.1186/s40780-025-00423-8 (PMC11900651; doi:10.1186/s40780-025-00423-8)
Supplement: Supplementary file 1 — Additional file 1. [file 40780_2025_423_MOESM1_ESM.docx]

Additional File: Fig. 1. Diagnostic plots of vancomycin concentrations

（A）

（B）

（C）

（D）

The observed versus population predicted vancomycin concentration (A), individual predicted vancomycin concentration (B), conditional weighted residuals vs. time after dosing (C), individual weighted residuals vs. individual predicted vancomycin concentration (D) are presented.

DV, observed concentration; PRED, predicted concentration; IPRED, individual predicted concentration; CWRES, conditional weighted residuals; IWRES, individual weighted residuals
