# Supplementary material for: Development and validation of a population pharmacokinetic model of vancomycin for patients of advanced age
Source: J Pharm Health Care Sci. 2025 Mar 12;11:18. doi: 10.1186/s40780-025-00423-8 (PMC11900651; doi:10.1186/s40780-025-00423-8)
Supplement: Supplementary file 3 — Additional file 3. [file 40780_2025_423_MOESM3_ESM.docx]

Additional File: Table 1. The equation of the Phoenix code used in the present study

| Model | Code |
| --- | --- |
| Additive model | Cobs = C + ε |
| Multiplicative model | Cobs = C × (1 +ε) |
| Additive plus multiplicative model | Cobs = C +ε × sqrt [1 + C^2^ × (Cmultstdev/σ)^2^] |

Cobs, served concentration; C, predictive concentration; ε, the error terms with a mean of 0 and a variance of σ^2^; Cmultstdev, multiplicative component of the residual; σ, standard deviation
