# Supplementary material for: Development and validation of a population pharmacokinetic model of vancomycin for patients of advanced age
Source: J Pharm Health Care Sci. 2025 Mar 12;11:18. doi: 10.1186/s40780-025-00423-8 (PMC11900651; doi:10.1186/s40780-025-00423-8)
Supplement: Supplementary file 4 — Additional file 4. [file 40780_2025_423_MOESM4_ESM.docx]

Additional File: Table 2. The equation used to estimate renal function in the present study

| **No. of equation** | **Equation** |
| --- | --- |
| **Equation 1^*^ (10)** | CLcr (male) = ([140 − age] × BW) / (SCr × 72) |
| **Equation 2^*^ (10)** | CLcr (female) = male × 0.85 |
| **Equation 3^*^ (17)** | eGFR (male) = 194 × SCr^-1.094^ × age^−0.287^ × body surface area (BSA)/1.73 |
| **Equation 4^*^ (17)** | eGFR (female) = male × 0.739 |
| **Equation 5 (18)** | BSA = 0.007184 × height (cm)^0.725^ × BW (kg)^0.425^ |

^*^ SCr was measured using an enzymatic method. CLcr, creatinine clearance; SCr, serum creatinine; eGFR, the glomerular filtration rate estimated from creatinine
