# Supplementary material for: Development and validation of a population pharmacokinetic model of vancomycin for patients of advanced age
Source: J Pharm Health Care Sci. 2025 Mar 12;11:18. doi: 10.1186/s40780-025-00423-8 (PMC11900651; doi:10.1186/s40780-025-00423-8)
Supplement: Supplementary file 5 — Additional file 5. [file 40780_2025_423_MOESM5_ESM.docx]

Additional File: Table 3. Population pharmacokinetic models for external evaluation in previous studies.

| Report | Population | Model |
| --- | --- | --- |
| Yasuhara et al. 1998 (8) | N = 190 (1,253 samples)  Age (years): 64.3 ± 13.8 ^*^  Body weight (kg): 52.3 ± 9.6 ^*^  CLcr (mL/min): 77.1 ± 50.9 ^*^ | CL (L/h) = 0.0478 × CLcr  Vss (L) = 60.7  K12 (/h) = 0.525  K21 (/h) = 0.213 |
| Oda et al. 2024 (7) | N = 7,167 (13,372 samples)  Age (years): 70.1 ± 16.5 *  Body weight (kg): 55.7 ± 14.8 *  CLcr (mL/min): 67.5 ± 40.1 * | CL (L/h) = 4.9 × (CLcr/120) ^0.959^  V1 (L) = 32.1 × (body wight/60) ^0.532^  Q (L/h) = 8.33  V2 (L) = 58.2× (body wight/60) ^0.348^ |

CLcr, estimated creatinine clearance; V1, central volume of distribution; V2, peripheral volume of distribution; Q, clearance between the central and peripheral compartments; V_SS,_ combined central and peripheral volumes of distribution at steady state; K12 and K21 transfer constant from central to peripheral and peripheral to central compartments, respectively. *mean ± standard deviation
