# Supplementary material for: Development and validation of a population pharmacokinetic model of vancomycin for patients of advanced age
Source: J Pharm Health Care Sci. 2025 Mar 12;11:18. doi: 10.1186/s40780-025-00423-8 (PMC11900651; doi:10.1186/s40780-025-00423-8)
Supplement: Supplementary file 6 — Additional file 6. [file 40780_2025_423_MOESM6_ESM.docx]

Additional File: Table 4. The equation used to determine the mean absolute prediction error and mean standard error

| Number of Equation | Equation |
| --- | --- |
| No. 1 | $Mean absolute prediction error=\frac{1}{n}\sum_{i=1}^{n} \left\vert{prediction}_{i}-{observation}_{i} \right\vert$ |
| No. 2 | $Mean standard error=\frac{1}{n}\sum_{i=1}^{n} {({observation}_{i}{-prediction}_{i})}^{2}$ |

n, total number of the dataset
