# Supplementary material for: Development and validation of a population pharmacokinetic model of vancomycin for patients of advanced age
Source: J Pharm Health Care Sci. 2025 Mar 12;11:18. doi: 10.1186/s40780-025-00423-8 (PMC11900651; doi:10.1186/s40780-025-00423-8)
Supplement: Supplementary file 7 — Additional file 7. [file 40780_2025_423_MOESM7_ESM.docx]

Additional File: Table 5. Clearance of vancomycin based on the creatinine clearance and serum albumin

|  | Clearance of vancomycin (L/h) | | | | | | | | | | | | | |
| --- | --- | --- | --- | --- | --- | --- | --- | --- | --- | --- | --- | --- | --- | --- |
| Alb  (g/dL) | CLcr (L/h)  [CLcr (mL/min)] | | | | | | | | | | | | | |
|  | 1.2  [20] | 1.5  [25] | 1.8  [30] | 2.1  [35] | 2.4  [40] | 2.7  [45] | 3.0  [50] | 3.3  [55] | 3.6  [60] | 3.9  [65] | 4.2  [70] | 4.5  [75] | 4.8  [80] | 5.1  [85] |
| 1.5 | 1.09 | 1.25 | 1.41 | 1.55 | 1.69 | 1.82 | 1.95 | 2.07 | 2.19 | 2.30 | 2.41 | 2.52 | 2.62 | 2.73 |
| 2.0 | 1.16 | 1.34 | 1.50 | 1.65 | 1.80 | 1.94 | 2.07 | 2.20 | 2.33 | 2.45 | 2.57 | 2.68 | 2.80 | 2.91 |
| 2.5 | 1.22 | 1.40 | 1.58 | 1.74 | 1.89 | 2.04 | 2.18 | 2.32 | 2.45 | 2.57 | 2.70 | 2.82 | 2.94 | 3.05 |
| 3.0 | 1.27 | 1.46 | 1.64 | 1.81 | 1.97 | 2.12 | 2.27 | 2.41 | 2.55 | 2.68 | 2.81 | 2.93 | 3.06 | 3.18 |
| 3.5 | 1.31 | 1.51 | 1.70 | 1.87 | 2.04 | 2.20 | 2.35 | 2.49 | 2.64 | 2.77 | 2.91 | 3.04 | 3.16 | 3.29 |

Alb, serum albumin; CL, clearance of vancomycin; CLcr, creatinine clearance
