# Supplementary material for: Development and validation of a population pharmacokinetic model of vancomycin for patients of advanced age
Source: J Pharm Health Care Sci. 2025 Mar 12;11:18. doi: 10.1186/s40780-025-00423-8 (PMC11900651; doi:10.1186/s40780-025-00423-8)
Supplement: Supplementary file 8 — Additional file 8. [file 40780_2025_423_MOESM8_ESM.docx]

Additional File: Table 6. Determination of the probability of target attainment based on the dosage and clearance of vancomycin

| VCM  daily dose (mg) | Determination of the probability of attaining an AUCss/MIC of ≥400 | | | | | | | | | | | | | |
| --- | --- | --- | --- | --- | --- | --- | --- | --- | --- | --- | --- | --- | --- | --- |
|  | CL (L/h) | | | | | | | | | | | | | |
|  | 0.5 | 0.75 | 1.0 | 1.25 | 1.5 | 1.75 | 2.0 | 2.25 | 2.5 | 2.75 | 3.0 | 3.25 | 3.5 | 3.75 |
| 250 | ○ | △ | △ | △ | △ | △ | △ | △ | △ | △ | △ | △ | △ | △ |
| 500 | ◎ | ○ | ○ | △ | △ | △ | △ | △ | △ | △ | △ | △ | △ | △ |
| 750 | ◎ | ◎ | ◎ | ○ | ○ | ○ | △ | △ | △ | △ | △ | △ | △ | △ |
| 1000 | ◎ | ◎ | ◎ | ◎ | ○ | ○ | ○ | ○ | △ | △ | △ | △ | △ | △ |
| 1250 | ◎ | ◎ | ◎ | ◎ | ◎ | ◎ | ◎ | ◎ | ◎ | ◎ | ○ | △ | △ | △ |
| 1500 | ◎ | ◎ | ◎ | ◎ | ◎ | ◎ | ◎ | ◎ | ◎ | ◎ | ○ | ○ | ○ | △ |
| 2000 | ◎ | ◎ | ◎ | ◎ | ◎ | ◎ | ◎ | ◎ | ◎ | ◎ | ○ | ○ | ○ | ○ |
| 2500 | ◎ | ◎ | ◎ | ◎ | ◎ | ◎ | ◎ | ◎ | ◎ | ◎ | ◎ | ○ | ○ | ○ |
| 3000 | ◎ | ◎ | ◎ | ◎ | ◎ | ◎ | ◎ | ◎ | ◎ | ◎ | ◎ | ◎ | ◎ | ◎ |

VCM, vancomycin; CL, clearance of vancomycin; AUCss, area under the concentration-time curve of vancomycin from 0 to 24 h at steady state; MIC, minimum inhibitory concentration; ◎, probability of AUCss/MIC ≥400 attainment ≥ 95%; ○, probability of AUCss/MIC ≥400 attainment ≥85–<95%; △, probability of AUCss/MIC ≥400 attainment < 85%
