# Supplementary material for: Development and validation of a population pharmacokinetic model of vancomycin for patients of advanced age
Source: J Pharm Health Care Sci. 2025 Mar 12;11:18. doi: 10.1186/s40780-025-00423-8 (PMC11900651; doi:10.1186/s40780-025-00423-8)
Supplement: Supplementary file 9 — Additional file 9. [file 40780_2025_423_MOESM9_ESM.docx]

Additional File: Table 7. Risk prediction of the probability of an AUCss of >600 µg・h/mL based on the dosage and clearance of vancomycin

| VCM  daily dose (mg) | Risk prediction of population probability of AUCss >600 µg・h/mL attainment | | | | | | | | | | | | | |
| --- | --- | --- | --- | --- | --- | --- | --- | --- | --- | --- | --- | --- | --- | --- |
|  | CL (L/h) | | | | | | | | | | | | | |
|  | 0.5 | 0.75 | 1.0 | 1.25 | 1.5 | 1.75 | 2.0 | 2.25 | 2.5 | 2.75 | 3.0 | 3.25 | 3.5 | 3.75 |
| 250 | ○ | ◎ | ◎ | ◎ | ◎ | ◎ | ◎ | ◎ | ◎ | ◎ | ◎ | ◎ | ◎ | ◎ |
| 500 | △ | △ | ◎ | ◎ | ◎ | ◎ | ◎ | ◎ | ◎ | ◎ | ◎ | ◎ | ◎ | ◎ |
| 750 | △ | △ | △ | △ | ◎ | ◎ | ◎ | ◎ | ◎ | ◎ | ◎ | ◎ | ◎ | ◎ |
| 1000 | △ | △ | △ | △ | △ | ○ | ◎ | ◎ | ◎ | ◎ | ◎ | ◎ | ◎ | ◎ |
| 1250 | △ | △ | △ | △ | △ | △ | △ | ◎ | ◎ | ◎ | ◎ | ◎ | ◎ | ◎ |
| 1500 | △ | △ | △ | △ | △ | △ | △ | △ | △ | ◎ | ◎ | ◎ | ◎ | ◎ |
| 2000 | △ | △ | △ | △ | △ | △ | △ | △ | △ | △ | △ | △ | ◎ | ◎ |
| 2500 | △ | △ | △ | △ | △ | △ | △ | △ | △ | △ | △ | △ | △ | △ |
| 3000 | △ | △ | △ | △ | △ | △ | △ | △ | △ | △ | △ | △ | △ | △ |

VCM, vancomycin; CL, clearance of vancomycin; AUCss, area under the concentration-time curve of vancomycin from 0 to 24 h at steady state; ◎, probability of AUCss >600 µg・h/mL attainment <10% (low risk); ○, probability of AUCss >600 µg・h/mL attainment from 10 to below 25% (moderate risk); △, probability of AUCss >600 µg・h/mL attainment ≥25% (high risk)
