# Supplementary material for: Development and validation of a population pharmacokinetic model of vancomycin for patients of advanced age
Source: J Pharm Health Care Sci. 2025 Mar 12;11:18. doi: 10.1186/s40780-025-00423-8 (PMC11900651; doi:10.1186/s40780-025-00423-8)
Supplement: Supplementary file 10 — Additional file 10. [file 40780_2025_423_MOESM10_ESM.docx]

Additional File: Table 8. Risk prediction of the probability of an AUCss of >600 µg・h/mL for the present nomogram based on the creatinine clearance and serum albumin

|  | Risk prediction of population probability of AUCss >600µg・h/mL attainment | | | | | | | | | | | | | |
| --- | --- | --- | --- | --- | --- | --- | --- | --- | --- | --- | --- | --- | --- | --- |
| Alb  (g/dL) | CLcr (L/h)  [CLcr (mL/min)] | | | | | | | | | | | | | |
|  | 1.2  [20] | 1.5  [25] | 1.8  [30] | 2.1  [35] | 2.4  [40] | 2.7  [45] | 3.0  [50] | 3.3  [55] | 3.6  [60] | 3.9  [65] | 4.2  [70] | 4.5  [75] | 4.8  [80] | 5.1  [85] |
| 1.5 | ◎ | △ | ◎ | ◎ | ◎ | ◎ | ◎ | ◎ | ◎ | ◎ | ◎ | ◎ | ◎ | ◎ |
| 2.0 | △ | ◎ | ◎ | ◎ | ◎ | ◎ | ◎ | ◎ | ◎ | ◎ | ◎ | ◎ | ◎ | ◎ |
| 2.5 | △ | ◎ | ◎ | ◎ | ◎ | ◎ | ◎ | ◎ | ◎ | ◎ | ◎ | ◎ | ◎ | ◎ |
| 3.0 | ◎ | ◎ | ◎ | ◎ | ◎ | ◎ | ◎ | ◎ | ◎ | ◎ | ◎ | ◎ | ◎ | ◎ |
| 3.5 | ◎ | ◎ | ◎ | ◎ | ◎ | ◎ | ◎ | ◎ | ◎ | ◎ | ◎ | ◎ | ◎ | ◎ |

Alb, serum albumin; CLcr, creatinine clearance; AUCss, area under the concentration-time curve of vancomycin from 0 to 24 h at steady state, ◎, probability of AUCss >600 µg・h/mL attainment <10% (low risk); ○, probability of AUCss >600 µg・h/mL attainment from 10 to below 25% (moderate risk); △, probability of AUCss >600 µg・h/mL attainment ≥25% (high risk)
